# Detection of endocrine disorders in young children with multi-transfused thalassemia major

**DOI:** 10.1186/s13052-021-01116-2

**Published:** 2021-07-31

**Authors:** Ramadan A. Mahmoud, Ashraf Khodeary, Marwa S. Farhan

**Affiliations:** 1 Department of Paediatrics, Faculty of Medicine, Sohag University, Sohag, 82524 Egypt; 2 Department of Clinical Pathology, Faculty of Medicine, Sohag University, Sohag, Egypt; 3 Department of Haematology and Clinical Pathology, Faculty of Medicine, Cairo University, Giza, Egypt

**Keywords:** Thalassemia major, Endocrine disorders, Young children, Iron chelating agent

## Abstract

**Background:**

Beta thalassemia major (TM) is the most common inherited genetic disorder worldwide. Patients are at risk of iron overload, which leads to various forms of tissue damage, including endocrinopathies. The aim of this study was to evaluate the prevalence and risk factors of endocrine disorders in young patients with multi-transfused TM receiving iron chelation therapy.

**Methods:**

The inclusion criteria included all known cases of TM according to hemoglobin electrophoresis data, aged 12 years or younger, during the study period. The patient’s age, gender, parent’s consanguinity, clinical examination, and types of iron chelating agents used were recorded. Serum ferritin level, complete blood count (CBC), blood glucose homeostasis, thyroid, and parathyroid functions were determined.

**Results:**

One hundred twenty patients met the inclusion criteria; 70% of them had malnutrition. The presence of endocrine disorders was observed in 28/120 (23.33%) patients. The most common endocrine disorders were thyroid disorders, either subclinical or clinical hypothyroidism in 11/120 (9.17%) patients, followed by abnormalities in glucose homeostasis 9/120 (7.5%). The prevalence of impaired glucose tolerance, impaired fasting glucose, and diabetes mellitus in the present study was 5 (4.17%), 4 (3.33%), and 0 (00%), respectively, while the least frequent endocrine disorder seen in our patients was hypoparathyroidism in 8/120 (6.66%). We noted that high serum ferritin levels and poor patient compliance to therapy were significantly associated with increased endocrine disorders (OR 0.98, 95% CI 0.96–0.99, *P* = 0.003 and OR 0.38, 95% CI 0.16:0.93, *P* = 0.03, respectively). Combined chelating iron agents significantly decreased the prevalence of endocrine disorders when compared with monotherapy (OR 0.40, 95% CI 0.16:0.97, *P* = 0.04).

**Conclusion:**

Endocrine disorders could occur in TM patients early before or equal to 12 years of life in about one-fourth of the patients. A high serum ferritin level and poor patient compliance to therapy were significantly associated with increased endocrine disorders. Combined iron-chelating agents were associated with a decreased prevalence of endocrine disorders when compared with monotherapy.

## Introduction

Beta thalassemia major (TM) is an autosomal recessive inherited disorder caused by decreased or absent β-globin chain production. There are 200 mutations linked with a TM phenotype that affect the stages of β-globin gene expression [1]. TM is the most prevalent monogenic disorder in the world, and the incidence rate is higher in the Middle East [2]. TM is the most common chronic hemolytic anemia in Egypt. It constitutes a major health problem with an estimated carrier rate of 9–10% [3].

TM brings long-term extravascular hemolysis, which increases iron absorption in the intestinal tract. Combined with multiple blood transfusions, this could lead to iron overload and an increased amount of iron in the tissue, which can cause progressive tissue damage in the liver, heart, endocrine glands, and other organs by generating hydroxyl free radicals and oxidative stress [4–6].

Treatment of TM is always individualized and modified according to the patient’s conditions. Conventional treatment consists of regular transfusions, an iron chelating agent, splenectomy, supportive therapies, and psychological support [7]. Non-conventional treatment includes hematopoietic stem cell transplantation, which remains the only curative treatment, fetal hemoglobin modulation, and gene therapy [8, 9]. Furthermore, the life expectancy and the quality of life of TM has improved remarkably over the last decades following optimized transfusion programs and improvements in chelation therapy [10, 11]. However, patients still experience a range of problems, particularly in relation to their growth, development, malnutrition, transfusion-transmitted infections, and tissue damage such as in the liver, heart, and endocrine system. These factors may contribute to the morbidity and mortality of these patients [12].

Endocrinopathies are common in patients with TM despite parenteral and oral iron chelation therapy. A majority of studies have focused on endocrine disorders in the adult population over 12 years of age. Many of these reports are from the Mediterranean area, and few studies have been conducted in children and young adults from Asian populations [13–17]. These studies researched the efficacy of TM treatments in preventing endocrine disorders. There are no studies in the literature describing the possibility of endocrine disorders that may start early in TM patients before 12 years of age. The aim of this study was to evaluate the prevalence of clinical and subclinical hypothyroidism, hypocalcemia, hyperphosphatemia, and impaired glucose metabolism in patients with TM who were 12 years of age or younger receiving oral or parental iron chelation therapy.

## Materials and methods

### Subject and data collection

In this cross-sectional study, we analyzed blood samples taken from 120 transfusion-dependent TM children age 12 years or younger during the period from May 2017 to May 2019 in the Pediatrics Departments, Faculty of Medicine, Sohag University, Egypt. Ethical approval for the study was obtained from the Research Committee of the Medical Faculty, Sohag University (Number 612, 2017), and written informed consent was obtained from all guardians/parents of the children prior to allocation of the children to the study.

The inclusion criteria included all known children with TM according to hemoglobin electrophoresis data aged 12 years or younger. Completed laboratory investigations were mandatory for inclusion the study. A clinical history and full examination were performed for each child included in the study. All patients were under a regular transfusion program with the aim of maintaining pre-transfusion hemoglobin levels above 9 g/dl. Hemophilic children and children with other types of hemolytic anemias, such as α-thalassemia, sickle cell anemia, and spherocytosis were excluded from the study.

A detailed history was taken with emphasis on personal history, parent consanguinity, family history of TM, family history of endocrine disorders, number of blood transfusions/month, and duration of disease, and a full clinical examination was performed, including anthropometric measurements, pallor, jaundice, hepatomegaly, cirrhotic manifestations, splenectomy, and cardiac examination. As regards the types of iron chelating agents used, we started with deferasirox as initial therapy when the serum ferritin level was greater than or equal to 1000 μg/L. However, if deferasirox was not available or ineffective, deferoxamine was used or added as adjuvant therapy according to the serum ferritin level.

For assessment of nutritional status, weight, length, body mass index (BMI) were reordered. World Health Organization (WHO) Z-score (standard deviation scores) for BMI (weight in kilograms divided by the square of height in meters) with using a cut-points of <− 2.0, > 1.0, > 2.0 and > 3.0 were used to classify malnutrition, risk of overweight, overweight, and obese, respectively [18].

### Laboratory investigations

Form all patients, 8 ml venous blood was obtained by venipuncture and was divided into two tubes, 2 ml blood in an EDTA vacutainer for complete blood count (CBC) and glycosylated hemoglobin (HbA1C) and 6 ml blood in two plane vacutainers for serum ferritin level, thyroid stimulating hormone (TSH), free thyroxine 3 (FT3), free thyroxine 4 (FT4), serum calcium (Ca), phosphate (P), alkaline phosphatase (ALP), parathyroid hormone (PTH).

All patients included in the study underwent both fasting blood glucose and oral glucose load tests. Glucose tolerance was classified into three categories based on the fasting blood glucose level: I) fasting blood glucose < 100 mg/ dL was considered normal; II) fasting blood glucose of 100 mg/dl and less than 126 mg/dL was defined as impaired fasting glucose; and III) a fasting blood glucose ≥126 mg/dL warranted the diagnosis of diabetes. Based on the oral glucose load test, impaired glucose tolerance was defined as a blood glucose level between 140 and < 200 mg/dL, and diabetes was defined as a glucose level ≥ 200 mg/ dL, 2 h after a 75-g oral glucose load. Individuals with impaired fasting glucose and/or impaired glucose tolerance were recently designated as pre-diabetic by the American Diabetes Association [19].

CBC was performed with a Celtac automated blood counter following calibration. HbA1C was assayed with a Misba i2 semi-automated device (Aggapy, India). Random blood glucose was estimated with a BT1500 fully automated chemistry analyzer (Biotechnica, Italy). Serum ferritin and thyroid hormones (FT3, FT4, and TSH) were measured by the Vitek Immuno Diagnostic Assay System (VIDAS- BioMerieux, France), an enzyme-linked fluorescent assay, according to the manufacturer’s instructions. Parathyroid hormone (PTH) was estimated with the IFlash1800 fully automated immunoassay system (Shenzhen Yhlo Biotech Co., Ltd.)

Primary overt hypothyroidism was diagnosed if FT3 was less than 2.50 pg/mL (normal range in children 2.50–4.64 pg/mL) or FT4 was less than 0.8 ng/dL (normal range in children 0.8–2 ng/dL) or both with high TSH over 6 mIU/ml (normal range 0.35–6 mIU/ml). Normal FT4 and FT3 and greater than normal TSH were considered evidence for subclinical hypothyroidism. Hypoparathyroidism was diagnosed if PTH was less than 15 pg/ml (normal range 15–65 pg/ml), hypocalcemia if less than 7 mg/dl (normal range 7–10 mg/dl), and hyperphosphatemia if more than 6.5 mg/dl (normal range 4.5–6.5 mg/dL). Increased alkaline phosphatase (ALP) if more than 450 U/L. (normal range 250 to 450 U/L).

### Statistical analysis

Data were analyzed using STATA intercooled version 12.1. Quantitative data were represented as mean, standard deviation, median, and range. Data were analyzed using a Mann–Whitney test as the distribution was not normal. Qualitative data were presented as number and percentage and compared using either a chi-square test or Fisher’s exact test. A binary logistic regression model was used to calculate the odds ratio (OR), 95% confidence interval (CI), and *P* value of the risk factors of endocrine disorders. Results were considered significant at *P* < 0.05.

## Results

Of 120 TM children, 54 (45%) patients were female and 66 (55%) were male. The mean (SD) patient age at the time of the study was 11.34 (2.27), range: 2–12 years and 11.12 (2.01) range: 2–12 in males and females, respectively. The mean (SD) duration of the disease was 5.74 (3.45) and 5.14 (3.20) in males and females, respectively. Patient’s anthropometric measurements, iron chelation therapy pattern and other patient characteristics are shown in Table 1.

**Table 1:** Patient’s characteristics of study population

Characteristics	Summary statistics Total 120 patients	
	Male 66 (55%)	Female 54 (45%)
Age / years		
Mean (SD)	11.34 (2.27)	11.12 (2.01)
Median (range)	11.4 (2–12)	11.6 (2–12)
Duration of disease/years		
Mean (SD)	5.74 (3.45)	5.14 (3.20)
Weight (Kg) Mean (SD)	28 (7.65)	25 (6.67)
Height (Cm) Mean (SD)	129 (19.54)	124 (18.35)
Body mass index (BMI) kg/m^2^		
Mean (SD)	16.82 (8.73)	16.26 (6.78)
Median (range)	16.5 (14–28)	16.9 (15–29)
Malnutrition according to Z score for BMI (percentage)	44 (66.67%)	40 (74.07%)
Family history of thalassemia major		
No	26 (39.39%)	20 (37.04%)
Yes	40 (60.61%)	34 (62.96%)
Family history of endocrine disorders		
No	41 (62.12%)	32 (59.26%)
Yes	25 (37.88%)	22 (40.74%)
Ferritin level		
Mean (SD)	3121 (1390)	2997 (1480)
Median (range)	2653 (58–6550)	2342 (47–6520)
Hemoglobin level before transfusion (Gram/DL)		
Mean (SD)	6.82 (1.45)	6.65 (1.89)
Median (range)	7.5 (2.8–11)	7.2 (2.5–11)
Splenectomy		
No	30 (45.45%)	35 (64.81%)
Yes	36(54.55%)	19 (35.19%)
Deferasirox (N. of patients)	20 (30.30%)	16 (29.63%)
Desferoxamine (N. of patients)	14 (21.22%)	11 (20.37%)
Combined therapy (N. of patients)	32 (48.48%)	27 (50%)

Most of the patients had no endocrine disorders (92/120, 76.67%; Table 2). The presence of any endocrine disorder was observed in 28/120 (23.33%) patients. However, the most common endocrine disorders were thyroid disorders 11/120 (9.17%) (subclinical hypothyroidism 6/120 [5.00%] and clinical hypothyroidism and 5/120 [4.17%]), followed by abnormalities in glucose homeostasis 9/120 (7.5%), and none of them had diabetes mellitus (DM). The least common endocrine disorders seen in our patients was hypoparathyroidism in 8/120 (6.66%). Hypocalcemia was found in 7/120 (5.83%), hyperphosphatemia in 10/120 (8.33%), and increased alkaline phosphatase in 13/120 (10.8%).

**Table 2:** The prevalence of endocrine disorders of studied population

Diseases	Number120 cases (percentage)
No endocrine disorders	92 (76.67%)
Impaired glucose tolerance (IGT)	5 (4.17%)
Impaired fasting glucose (IFG)	4 (3.33%)
Diabetes mellitus (DM)	0 (00%)
Subclinical hypothyroidism	6 (5.00%)
Clinical hypothyroidism	5 (4.17%)
Hypoparathyroidism	8 (6.66%)

**Table 3:** Risk factors of endocrine disorders in young children with thalassemia major

	Endocrine disorder (28/120)	No endocrine disorder (92/120)	Odds ratio (95% confidence interval)	*P* value
Age mean (SD)	11.60 (3.21)	11.01 (1.27)	1.33 (0.48:2.21)	0.90
Gender				
Females	12 (42.86%)	42 (45.65%)	1	
Males	16 (57.14%)	50 (54.35%)	1.12 (0.48:2.63)	0.80
Duration of the disease/years Mean (SD)	6.22 (1.20)	5.28 (2.64)	1.20 (0.98:1.45)	0.07
Malnutrition				
No	12 (42.86%)	24 (26.09%)	1	
Yes	16 (57.14)	68 (73.91)	0.47 (0.19:1.14)	0.09
Hemoglobin level before blood transfusion (Gram/DL) Mean (SD)	7.12 (1.62)	6.69 (1.89)	1.14 (0.90:1.46)	0.29
Splenectomy				
No	13 (46.43%)	52 (56.52%)	1	
Yes	15 (53.57%)	40 (43.48%)	1.50 (0.64:3.50)	0.35
Family history of thalassemia major				
No	13 (46.43%)	33 (35.87%)	1	
Yes	15 (53.57%)	59 (64.13%)	0.64 (0.27:1.51)	0.65
Family history of endocrine disorders				
No	15 (53.57%)	58 (63.04%)	1	
Yes	13 (46.43%)	34 (36.96%)	1.48 (0.63:3.48)	0.37
Ferritin level Mean (SD)	3631.45(1636)	2658.40 (1443)	0.98 (0.96–0.99)	0.003
Deferasirox				
No	16 (57.14%)	68(73.91%)	1	
Yes	12 (42.86%)	24 (26.09%)	2.13 (0.88:5.13)	0.09
Desferoxamine				
No	21 (75.00%)	74 (80.43%)	1	
Yes	7 (25.00%)	18 (19.57%)	1.37 (0.50:3.72)	0.54
Combined therapy				
No	19 (67.86%]	42 (45.65%)	1	
Yes	9 (32.14%)	50 (54.35%)	0.40 (0.16:0.97)	0.04
Compliance to medication				
Received ≤50%/month	19 (67.86%]	41(44.57%	1	
Received > 50/month	9 (32.14%)	51 (55.43%	0.38 (0.16:0.93)	0.03

*P* value was obtained from univariate logistic regression

As shown in Table 3, the presence of endocrine disorders in the studied population was not significantly associated with the risk factors age, gender, hemoglobin level before transfusion, or splenectomy status (*P* > 0.05). Moreover, there were no significant differences between the mean (SD) duration of TM in patients with endocrine disorders at 6.22 (1.20) years compared with non-endocrine disorders at 5.28 (2.64) years (OR 1.20, 95% CI 0.98:1.45, *P* = 0.07). However, there were a significant increase in the mean (SD) serum ferritin levels in the studied TM patients with endocrine disorders 3631.45 (1636) μg/L when compared with the studied TM patients without endocrine abnormalities 2658.40 (1443) μg/L (OR 0.98, 95% CI 0.96–0.99, *P* = 0.003).

Regarding iron chelating therapy, there were no significant change between patients with or without endocrine disorders regard monotherapy started with deferasirox or desferoxamine. However, in those who received combined therapy, 9 of 28 (32.14%) cases had endocrine disorders, compared with 50 of 92 (54.35%) cases who did not have endocrine disorders, (OR 0.40, 95% CI 0.16:0.97, *P* = 0.04; Table 3).

There were 84 of 120 (70%) cases who suffered from malnutrition with Z score < − 2 for BMI; Table 1, However, there was no significant differences between TM in those who had endocrine disorders and those who did not have endocrine disorders regarding malnutrition (OR 0.47, 95% CI 0.19:1.14), (*P* = 0.09). We found poor compliance with iron chelating agents, in which 19 of 28 (67.86%) in the endocrine disorder group received less than 50% of the calculated doses per month, compared with 41 of 92 (44.57%) in the non-endocrine disorder group (OR 0.38, 95% CI 0.16:0.93, *P* = 0.03; Table 3).

## Discussion

The life expectancy of patients with TM has increased due to improved therapeutic options [5]. However various complications such as endocrinopathies, cardiomyopathies, and bone disorders are still obstacles to treatment in these patients [4–6]. In the present study, we noted that endocrinopathies appear early in approximately one fourth of TM patients at the age of 12 years or younger. A high serum ferritin level is usually associated with increased endocrinopathies. Combined iron-chelating agents were associated with a decreased prevalence of endocrine disorders when compared with monotherapy.

In the present study, we report that an increased serum ferritin level is associated with increased endocrine disorders in TM patients. A high prevalence of endocrine abnormalities in TM patients associated with increased iron overload was previously reported [3, 5, 10, 20]. However, other reports did not find a significant relationship between endocrine disorders and serum ferritin level [21, 22].. The serum ferritin level could estimate the liver iron concentration in TM [23]. Moreover, Sobhani et al. [24] found that higher serum ferritin strongly predicted the severity of cardiac and liver iron overload.

In this study, two thirds of the cases had malnutrition. We defined malnutrition according to WHO definition by Z score for BMI if < − 2, which was the most accepted definition worldwide [18]. Our cases’ BMIs were compatible with those reported by Biswas et al. [25], who studied 328 TM patients, approximately 37.2% of which were between 11 and 12 years of age. They found that 48.2% were malnourished with a mean BMI of 13.9 ± 1.6 kg/m^2^. TM has a significant impact on the growth process of children, and hence early intervention is needed to reduce the prevalence of malnutrition as good nutrition with adequate vitamin and trace element intake, along with calcium and vitamin D supplementation, side by side with optimal iron chelating therapy [26].

Early diagnosis and treatment of TM complications are essential to ensure a good quality of life and to reduce early morbidity and mortality. In TM patients with endocrine disorders, sometime early therapy is recommended in the form of thyroxine therapy. An improvement of subclinical hypothyroidism and primary hypothyroidism has been observed after intensive iron chelation therapy [17]. Therefore, from these data, periodic assessment of iron overload and follow-up of endocrine functions beside improved adherence to chelation therapy should be strongly considered in hospitals in which there are no electronic files or routine follow up of these patients, in order to improve their quality of life and life expectancy.

Iron chelation therapy is the only method of iron overload control in transfusion-dependent patients. In the present study, we found that oral deferasirox is not superior to deferoxamine when used as monotherapy. These data not agree with other reports [27, 28] showing that long-term oral deferasirox therapy may decrease the prevalence of endocrinopathies in TM. The main explanation for this may be the short duration of therapy in our study and the different age groups. However, in our study, combined therapy was associated with a decreased incidence of endocrine disorders, similar to data previously reported by Farmaki et al. [16]. Moreover, Ho et al. [29] found that combined therapy with deferoxamine and oral deferasirox improved clinical outcomes and quality of life in terms of iron chelation in transfusion-dependent patients with TM at a reasonable cost from a healthcare perspective. The TM patients in the present study had poor compliance to iron-chelating medication; this could explain the occurrence of early endocrine disorders. A similar result was previously reported by Sobhani et al. [24], who found that irregular use of chelating drugs was associated with a higher risk of iron tissue damage, regardless of the type of chelating agent.

In young children and adults, type 2 diabetes develops over a long period of time, often presenting initially as a pre-diabetic state such as impaired fasting glucose (IFG) or impaired glucose tolerance (IGT). Prediabetes raises the short-term absolute risk of type 2 diabetes by 3-to 10-fold, with some populations exhibiting greater risk than others [30]. Furthermore, pre-diabetic states are also at increased risk of cardiovascular disease [31]. Endocrine abnormalities should be monitored carefully, and a thorough endocrine evaluation should be carried out yearly in every patient with TM to detect subclinical endocrinopathies [26]. Early identification and treatment of persons with prediabetes has the potential to reduce or delay the progression to diabetes [32].

The total prevalence of abnormal glucose homeostasis in the present study was 7.50%, which could be divided into impaired glucose tolerance (IGT), impaired fasting glucose (IFG), and DM of 5 (4.17%), 4 (3.33%), and 0 (00%), respectively. In previous reports, the prevalence of DM was reported to vary between 0.00% [33] and 26.70% [20]. In a meta-analysis conducted by He LN et al. [34] including 35 studies, it was revealed that the prevalence of IGT, IFG, and DM in TM were 12.46, 17.21%, and 6.54, respectively, in patients aged from 2 to 28 years. The increased prevalence of abnormal glucose homeostasis in the previous meta-analysis [34] could be due to the different age groups. The prevalence of abnormal glucose homeostasis in TM was higher compared to that in non-TM children and young adults, which was 8–17 per 100,000 per year in one cohort [35].

We reported thyroid disorder in the present study at a rate of 9.17%. However, the overall prevalence of hypothyroidism in TM is variable between studies with different age groups; it was (14.6%) in Iran [36] and 21.6% in Italy [37]. Different results could be due to genetic, geographical, cultural, and economic factors and also due to the quality of blood transfusions and chelators. Our data demonstrated that the prevalence of hypothyroidism (9.17%) increased in TM patients when compared with non-TM children with sufficient dietary iodine, in which only 0.3–0.4% of children had hypothyroidism, and approximately 4.3–8.5% had subclinical hypothyroidism [38]. Moreover, the prevalence of hypoparathyroidism in our study was (6.6%) which is comparable with that in other studies with different age groups. In Saffari et al. [13] in TM patients with a mean (SD) age 21.26 ± 4.53 years, the prevalence of hypoparathyroidism was 7.79%, and in De Sanctis et al. [37] in TM patients with the mean age less than 16 years, the prevalence of hypoparathyroidism was 6.9%.

In our study, we tried to detect endocrine disorders in young age children with TM. However, we did not find any relationship between the duration of TM and endocrine disorders. This could be explained by other factor such as serum ferritin level and patient compliance with cheating therapy having a greater role than disease duration. Furthermore, we did not find a relationship between children with and without endocrine disorders in relation to family history of TM, family history of endocrine disorders, splenectomy, gender, or hemoglobin level before transfusion, in agreement with previously published studies [10, 20].

The presented study had some limitations; liver iron concentration determined by noninvasive magnetic resonance imaging relaxation time techniques was used to estimate body iron load and has been shown to predict total body iron stores more accurately than the serum ferritin level in some reports [39–41]. In this study, we measured iron overload from the serum ferritin level as liver iron concentration measurement was not available in our unit. We did not perform a follow up for the cases, as the purpose of this cross-sectional study was to take a picture of the prevalence of endocrinopathies and the risk factors associated with them in this population. We selected this age group because older age groups had already been studied extensively. Serial measurements of other endocrine functions such as growth hormone, reproductive hormones, and adrenal hormones in TM patients, needed in further studies.

## Conclusion

We recommended that endocrine evaluation in thalassemic patients be carried out routinely, even at a younger age, especially in patients with iron overload and poor compliance with chelating therapy, particularly for glucose homeostasis, hypothyroidism, and hypoparathyroidism. One fourth of studied patients had endocrine disorders, which were related to high serum ferritin levels. We reported that oral deferasirox is not superior to deferoxamine when used as monotherapy. However, combined iron-chelating agents were associated with a decreased prevalence of endocrine disorders when compared with monotherapy.

## Data Availability

The datasets used and/or analyzed during the current study are available from the corresponding author on reasonable request.

## Abbreviations

ALP: Alkaline phosphatase
BMI: Body mass index
Ca: Serum calcium
CBC: Complete blood count
CI: Confident interval
DM: Diabetes mellitus
FT3: Free thyroxine 3
FT4: Free thyroxine 4
HbA1C: Glycosylated hemoglobin
IFG: Impaired fasting glycose
IGT: Impaired glycose tolerance
OR: Odds ratio
P: Phosphate
PTH: Parathyroid hormone
TM: Thalassemia major
TSH: Thyroid stimulating hormone
WHO: World health organization
